# Distinct Metabolomic and Proteomic Signatures of Early Brain Damage Triggered by the Entrance Plateau Versus the Spread-Out Bragg Peak of Proton Radiation

**DOI:** 10.3390/cells15151382

**Published:** 2026-07-30

**Authors:** Keman Liao, Fei Xu, Yunsheng Gao, Xuming Jiang, Yingying Lin, Jiayi Chen

**Affiliations:** 1 Department of Radiation Oncology, Ruijin Hospital, Shanghai Jiaotong University School of Medicine, Shanghai 200025, China; kmliao@alumni.sjtu.edu.cn (K.L.); xf11756@rjh.com.cn (F.X.); gys11856@rjh.com.cn (Y.G.); jxm40919@rjh.com.cn (X.J.); 2 Shanghai Key Laboratory of Proton Therapy, Shanghai APACTRON Particle Equipment Co., Ltd., Ruijin Hospital, Shanghai Jiaotong University School of Medicine, Shanghai 201801, China; 3 Institute for Medical Imaging Technology, Shanghai United Imaging Intelligence Co., Ltd., Ruijin Hospital, Shanghai Jiaotong University School of Medicine, Shanghai 201800, China

**Keywords:** proton therapy, radiation-induced brain injury, entrance plateau, spread-out bragg peak

## Abstract

Proton therapy spares normal tissues better than photon therapy, potentially reducing toxicity while maintaining tumor control. However, challenges remain due to variations in proton dose distribution, particularly at the distal edge of the spread-out Bragg peak (SOBP); for organs at risk, such variation is crucial. We evaluated the biological effects by comparing two positions of the proton profile, the entrance plateau (EP) and SOBP, in a murine model and investigated distinct metabolomic and proteomic signatures. Mice exposed to the EP beam segment (LET_d_ = 0.8 keV/µm) exhibited less weight reduction than their SOBP-irradiated counterparts (LET_d_ = 2.6 keV/µm). Two hours post-irradiation, the SOBP caused more severe DNA damage in the hippocampus and thalamus. Hematoxylin and eosin staining revealed eosinophil aggregation in both groups, with more surviving neurons in the EP group. Metabolomic profiles differed more between the EP and SOBP groups at 2 h than at 3 days post-irradiation. Relative to EP, SOBP irradiation at 2 h increased fructose-1,6-bisphosphate (FBP), dihydroxyacetone phosphate (DHAP), and inosine but decreased prostaglandin F2α; subsequently, proteomic analysis at day 3 showed that calcium signaling, NF-κB, and endocytosis pathways were enriched in the SOBP group. Combined multi-omics analysis further demonstrated that SOBP irradiation significantly activated the pentose phosphate pathway, purine metabolism, and phospholipase D signaling, while concurrently suppressing arachidonic acid metabolism. Our findings underscore the need for early detection of proton-induced brain toxicity and demonstrate that the higher-LET SOBP segment causes more severe damage than the EP. Targeting these dysregulated multi-omics pathways may offer a promising strategy for mitigating radiation-induced brain injury during proton therapy.

## 1. Introduction

Proton therapy offers a distinct dosimetric advantage over conventional photon-based radiotherapy by depositing the majority of its energy at the Bragg peak, thereby sparing proximal and distal normal tissues [1,2,3]. This unique property particularly attractive for treating radioresistant tumors that require high-dose irradiation while minimizing damage to surrounding critical structures, including sarcomas, pediatric malignancies, uveal melanomas, and recurrent tumors requiring re-irradiation [4,5,6,7]. Despite these well-established dosimetric benefits, accumulating clinical evidence indicates that proton therapy is not devoid of adverse effects [8,9]. For example, optic nerve damage remains a significant and clinically challenging complication in uveal melanoma patients treated with proton beams, suggesting that the biological consequences of proton irradiation extend beyond simple physical dose distribution [10]. More fundamentally, the radiobiological effects along different segments of the proton beam path-specifically, the entrance plateau (EP), where linear energy transfer (LET) is relatively low, and the spread-out Bragg peak (SOBP), where LET is substantially elevated-remain incompletely characterized. These segment-specific biological responses may differentially contribute to treatment-associated toxicities and are not adequately captured by current treatment planning systems that rely primarily on physical dose metrics [11,12]. Therefore, a comprehensive understanding of the differential biological effects induced by EP and SOBP irradiation is urgently needed to optimize proton therapy, mitigate normal tissue injury, and ultimately improve the therapeutic ratio for patients undergoing this advanced modality.

The radiobiological effects of proton irradiation—that is, the cellular and molecular responses triggered by proton energy deposition—encompass a diverse array of mechanisms, including the induction of DNA double-strand breaks, the generation of reactive oxygen species and subsequent oxidative stress, the activation of inflammatory cascades [13,14], the perturbation of metabolic homeostasis, and the initiation of apoptotic or other cell death pathways. A critical determinant of these biological responses is linear energy transfer (LET), which describes the rate of energy deposition along the particle track [15]. Protons with higher LET produce dense ionization tracks, leading to more complex and less reparable DNA damage, and consequently confer higher relative biological effectiveness (RBE). Along the proton beam path, LET is not uniform: the entrance plateau (EP) is characterized by relatively low LET, whereas the spread-out Bragg peak (SOBP)—the region intentionally broadened to cover the tumor volume—exhibits substantially elevated LET. This LET heterogeneity raises the critical question of whether normal tissues traversed by different beam segments sustain distinct radiobiological damage, even when physical doses are comparable. This concern is supported by clinical observations of increased rib fracture rates and contrast-enhancing brain lesions at the distal margins of proton beams [8,9], regions associated with elevated LET. Furthermore, experimental studies have demonstrated that the center and distal edge of the SOBP elicit differential biological responses [11,12], underscoring the biological heterogeneity within the SOBP itself. Nevertheless, the biological effects of EP irradiation on normal tissues remain poorly characterized [16], representing a significant knowledge gap given that these tissues are inevitably irradiated along the beam path. With the rapid global expansion of proton therapy and the growing patient population [17,18], there is an urgent need for systematic in vivo investigations to characterize the differential radiobiological effects of EP and SOBP irradiation, particularly on late-responding normal tissues that often limit therapeutic dose escalation. To address this gap, the present study was designed to directly compare the biological responses to EP (LET_d_ = 0.8 keV/µm) and SOBP (LET_d_ = 2.6 keV/µm) irradiation in a murine model of radiation-induced brain injury, employing integrated metabolomic and proteomic approaches to delineate the underlying molecular mechanisms and identify segment-specific signatures of tissue damage.

Historically, single-omics analysis techniques have been employed to learn more about biological processes and distinguish between different groups. However, these approaches are frequently restricted to particular data types and levels. Multi-omics, including proteomics and metabolomics, may now be integrated to better understand the molecular mechanisms and genetic changes in microenvironment signatures in biological processes [19]. Notably, this method supports biological pathways by offering stronger evidence and enabling a comprehensive view of identified factors. For example, the integrated analysis of metabolomics and proteomics found 38 druggable proteins for atrial fibrillation and heart failure and linked these to 35 plasma metabolites relevant to cardiac disorders [20]. Additionally, the metabolic targets of a natural substance called Theaflavin-3′-gallate and its interactions within the HK2/TIGAR/MAPK pathway were identified by integrating non-targeted metabolomics and proteomics [21]. Hence, metabolomics and proteomics will contribute to unraveling the its intricate network of interactions within cellular pathways, holding promise for therapeutic interventions in the context of EP and SOBP proton irradiation.

In this work, we established a murine model of radiation-induced brain injury (RIBI) to investigate the early biological effects along the proton beam during dose delivery. Initially, acute radiation-induced damage was evaluated via weight loss and morphological observation. Next, we performed metabolomic and proteomic analyses on brain tissues treated with the same dose of EP and SOBP irradiation. We aimed to (1) investigate whether different segments of the proton beam cause varying radiobiological effects and (2) identify early, unique metabolomic and proteomic signatures after irradiation in vivo.

## 2. Materials and Methods

### 2.1. Animals

The study employed male and female C57BL/6 mice, 6–8 weeks of age and weighing 20–22 g, obtained from GemPharmatech in Jiangsu, China. The research team has undergone specialized training in handling and caring for animals. Ear punches and numbered cages were used to identify the animals. Each cage had a maximum of four mice, and cage positions were randomly arranged and regularly rotated to avoid location bias. The mice were all housed in a quiet, pathogen-free environment, provided with plenty of food and water, and exposed to a 12 h light–dark cycle. In order to preserve baseline conditions between groups, animals were divided based on age and body weight.

The mice were divided into experimental groups at random. Three experimental groups (*n* = 15/group) were established as follows: (1) non-irradiated, anesthetized sham controls; (2) EP proton-irradiated mice; and (3) SOBP proton-irradiated mice. Anesthesia was induced by an intraperitoneal injection of sodium pentobarbital (10 mg/mL, Yiheng, China) at a dosage of 50 mg/kg. The mice were exposed to 6 Gy (RBE) protons and followed up at different time points at 2 h, 1 day, and 3 days, and their weight was recorded before and after irradiation. If EP/SOBP-treated mice lost more than 20% of their body weight, they were promptly put to death by progressively raising the CO_2_ concentration in the anesthetic chamber.

Every experiment used a double-blind design. Yingying Lin, the principal investigator, knew how the groups were assigned at each stage of the mouse experiments. Throughout the entire experimentation, animal handling, and data analysis process, Keman Liao and the other researchers were blinded to the mouse group assignments. The animal study protocol was approved by Ruijin Hospital of Shanghai Jiaotong University School of Medicine approved all experimental procedures (approval code: RJ2024049 and 29 September 2024, date of approval).

### 2.2. Dose Delivery

**Figure 1:**
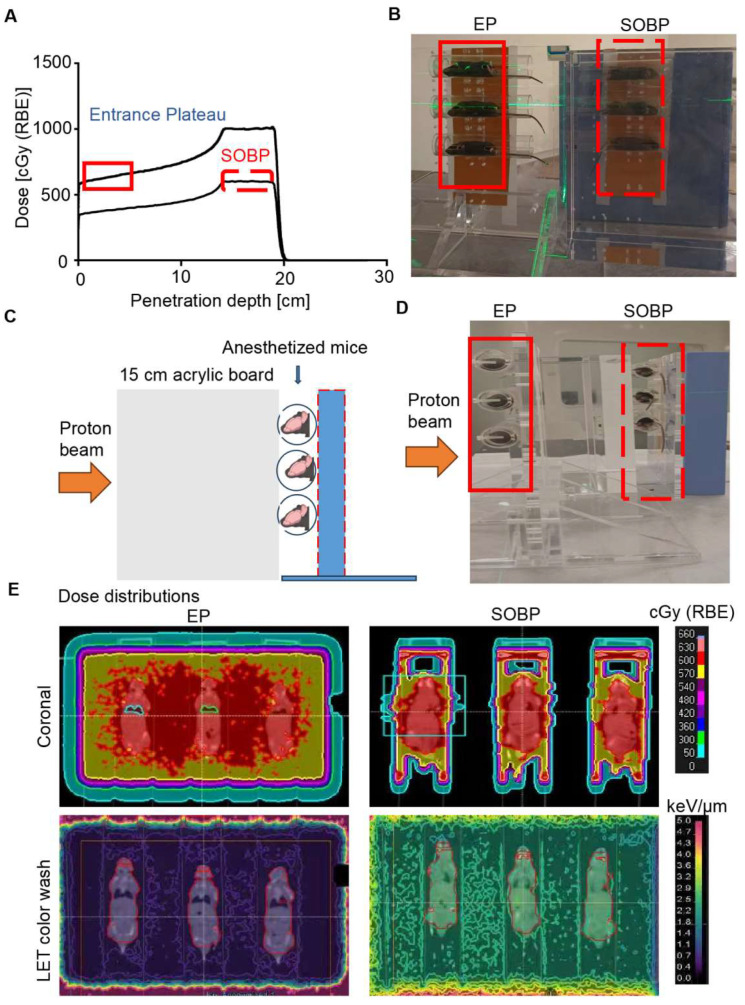
Construction of SOBP and EP mouse models with early brain toxicity under IR. (**A**) Schematic illustration of dose-length curves of SOBP and EP proton irradiation. (**B**) Position of mice under laser guidance during proton horizontal irradiation with 20 × 10 cm radiation field for EP section and SOBP section. (**C**) Schematic illustration of SOBP position. (**D**) Side view of mice under laser guidance during proton irradiation. (**E**) Dose distribution and LET color wash of EP and SOBP proton irradiation in coronal planes.

All irradiations were performed with proton pencil beam scanning (PBS) at the fixed horizontal proton beam line at the Shanghai Key Laboratory of Proton-Therapy. As shown in Figure 1 and Figure S1, the mice were anesthetized and placed in a mouse fixation device. For EP proton irradiation, three mice were placed in the mouse device with a radiation field of 20 cm × 10 cm and irradiated with a single dose of 6 Gy (RBE) ±3%. For SOBP proton irradiation, a 15 cm acrylic board was placed in front of the mouse device as a range shifter to ensure appropriate dose distribution across the mice. The SOBP radiation field is 20 cm × 10 cm with a plateau width of 5 cm to deliver a single dose of 6 Gy (RBE) ±3%. Both EP and SOBP groups were irradiated simultaneously, and 141.8–170.4 MeV pencil beams were scanned using the Raystation treatment planning system (version 10, RaySearch, Stockholm, Sweden). Regarding the positioning, the vertical laser line was aligned with the midpoint of the irradiated area, and the horizontal laser line was aimed at the middle mouse under anesthesia. The Z-axis laser line was aimed at the surface of the acrylic board for the SOBP group and the surface of the mouse device for the EP group (Figure S1A).

Temperature, pressure, polarity effect, and recombination corrections were made in accordance with the International Atomic Energy Agency’s TRS-398 Code of Practice (TRS-398, Technical Reports Series No. 398, International Atomic Energy Agency, Vienna, Austria, 2024) [22]. Proton irradiation dosage measuring points were placed in the middle of each group.

### 2.3. LET Calculation

According to the RayStation reference manual, LET from primary and secondary protons is included. The LET_d_ in a voxel i from a single beam b is computed as

(1) $${LETd}_{b,i}=\frac{\sum_{s,h}{\left(d*let\right)}_{i}}{D_{b,i}^{p}}$$

(2) $$D_{b,i}^{p}=\sum_{s,h}d_{i}$$

(3) $${\left(d*let\right)}_{i}=\frac{1}{\rho V}\int_{\Gamma_{i}}{LET}_{\infty }dE$$

where $D_{b,i}^{p}$ is the total beam dose, di is the dose deposited in voxel i from the Monte Carlo simulated proton h in spot s, and (d * let)_i_ is the integral of LET with respect to deposited energy along the proton track $\Gamma_{i}$ across the voxel. ρ and V are the mass density and volume of the voxel, respectively, and ${LET}_{\infty }$ is the unrestricted linear energy transfer in water. The E in Equation (3) is the energy of the proton only considering energy loss to the electrons along the $\Gamma_{i}$, and the energy threshold is 1 keV. The total LET_d_ from all beams is

(4) $${LET}_{d_{i}}=\frac{\sum_{b}D_{b,i}^{p}*{LETd}_{b,i}}{\sum_{b}D_{b,i}^{P}}$$

### 2.4. Section Preparation, Immunofluorescence, Hematoxylin and Eosin (H&E) Staining, and Nissl Staining

Male mouse brain tissues were fixed in paraformaldehyde at 2 h after radiation and sectioned at a thickness of 5 µm on glass slides. The primary antibodies used for immunofluorescence staining were γ-H2AX (1:200, Abcam, Cambridge, MA, USA; ab26350). Following incubation, slices underwent PBS washing, DAPI counterstaining, and examination under a Leica DM4B fluorescent microscope (Leica Microsystems, Wetzlar, Germany).

After five minutes of H&E staining, paraffin-embedded sections were washed with running water and then subjected to a three-minute eosin counterstaining phase.

After being dehydrated for one minute with 100%, 95%, and 70% alcohol, the brain slices were cleaned with ultrapure water. Following a half-hour staining with 0.1% tar violet, the tablets were cleaned with ultrapure water and then dehydrated for 1–2 min with 75.0%, 85.0%, 95.0%, and 100% alcohol. Lastly, the slices were cleared in xylene three times in a span of two minutes. Under a light microscope, the slices were sealed with neutral glue, and the number of neurons was counted.

### 2.5. Nontargeted Metabolite Profiling and Bioinformatics

Male mouse brain tissues from the EP and SOBP groups were harvested 2 h after proton irradiation and processed for LC-MS analysis. One aliquot was eluted from the T3 column (Waters ACQUITY Premier HSS T3 Column, 1.8 µm, 2.1 mm × 100 mm; Waters, Milford, MA, USA) using the gradient below, using 0.1% formic acid in water as solvent A and 0.1% formic acid in acetonitrile as solvent B. Positive ion conditions were used for the analysis: 5 to 20% in 2 min, 60% for 3 min, and 99% for 1 min, holding for 1.5 min before going back to 5% mobile phase B for 0.1 min and holding again for 2.4 min. The analytical parameters were as follows: a 40 °C column temperature, 0.4 mL/min flow rate, and 4 μL injection volume. A second aliquot that matched the elution gradient of the positive mode was used under negative ion conditions.

Each method alternated between full-scan MS and data-dependent MSn scans with dynamic exclusion. MS analyses utilizing electrospray ionization in both the positive and negative ion modes were carried out using full-scan analysis over *m*/*z* 75–1000 at 35,000 resolution.

The principal component analysis (PCA) was carried out in an unsupervised manner. Heatmaps with dendrograms were used to show the outcomes of the hierarchical cluster analysis (HCA) of the samples and metabolites. Differential metabolites were identified for the two-group study using VIP (VIP > 1) and *p*-value (*p*-value < 0.05, Student’s *t*-test). VIP values were extracted from the OPLS-DA results. The detected metabolites were annotated using KEGG Compound.

### 2.6. Proteomics and Bioinformatics

Three days after proton irradiation, brain tissues from male mice irradiated with EP and SOBP proton beams were harvested for LC-MS analysis. DIA-MS analysis was performed using an Orbitrap Astral mass spectrometer (Thermo Fisher, Waltham, MA, USA) coupled with a Vanquish Neo nano-LC system (Thermo Fisher). Positive ion mode was used with a full MS scan range of 380–980 *m*/*z* at 240,000 resolution (200 *m*/*z*), AGC target 500%, and maximum IT 5 ms. DIA acquisition included 299 windows (isolation window 2 Th), 25% HCD energy, 500% AGC, and 3 ms maximum IT 3. MS raw data was evaluated using DIA-NN (v1.8.1) without a library. *p*-values < 0.05 and FC ≥ 1.5 or FC ≤ 0.6667 were used to identify differential proteins for the two-group analysis. The discovered proteins were annotated using uniprotkb_proteome_UP000000589_mouse_54910_20240528.

### 2.7. Immunoblot Analysis

Proteins were extracted from the brain tissues of male and female mice using RIPA buffer. The denatured proteins were resolved by 10% or 15% SDS-PAGE. The proteins were next transferred to PVDF membranes (0.22 μm/0.45 μm, Millipore, Merck KGaA, Darmstadt, Germany), blocked with a blocking buffer (Beyotime, Shanghai, China, P0252), and incubated with primary antibodies: anti-Cox2 (1:1000, Proteintech, Wuhan, China, 27308-1-AP), anti-PDE1C (1:1000, Proteintech, Wuhan, China, 13785-1-AP), anti-FGFR2 (1:500, Proteintech, Wuhan, China, 13042-1-AP), anti-γ-H2AX (1:1000, Abcam, Cambridge, MA, USA; ab26350), anti-CAMKII (1:500, Proteintech, Wuhan, China, 12666-2-AP), and anti-ACTIN (1:10,000, Proteintech, Wuhan, China, 66009-1-Ig). Then, they were incubated with secondary antibodies and washed with TBST. The bands were visualized using an ECL chemiluminescence kit (Changzhou, China).

### 2.8. ELISAs

Serum from different groups of male and female mice was collected after cultivation periods of 2 h or 3 d. Two hours before supernatant collection, the blood samples were placed at room temperature. They were centrifuged for 20 min at 1000× *g* to collect the supernatant. The PGF2α content of the supernatant was analyzed using a Mouse PGF2α ELISA (Finetest, Wuhan, China; EM1284). The FBP content of the supernatant was analyzed using Mouse FBP ELISA (Huabang Bio, Hebei, China; HB-P9S3227X). An ELISA was conducted following the manufacturer’s instructions.

### 2.9. Statistics

IBM Corporation’s SPSS 21.0 software, based in Armonk, NY, USA, was used for statistical analysis. One-way analysis of variance (ANOVA) was used to identify group differences. Variance homogeneity was assessed before multiple comparisons. For datasets with equal variances, Tukey’s post hoc test was used. A two-tailed *p*-value of less than 0.05 was considered statistically significant. GraphPad Prism 7 (GraphPad Software Inc., San Diego, CA, USA) was used to produce the graphical representations.

## 3. Results

### 3.1. Establishment of Murine RIBI Model Under EP or SOBP Segment Irradiation

To create two irradiated areas at the same time, we split two equal beams of 6 Gy (RBE) for a whole-body irradiation model in EP and SOBP segments (Figure 1A). Anesthetized mice were placed in a fixed device to adjust their position under laser line guidance (Figure 1B–D). The dose distributions in the coronal planes were designed and verified via mouse CT (Figure 1E).

To imitate dose levels at the middle surfaces of the mice, a spot was placed at the corresponding heights of the middle mice in both the EP and SOBP groups. In the SOBP group, the measured equivalent depths were 15 cm on an acrylic board. The detected IR dose in the SOBP proton was 6.204 Gy (RBE). In the EP group, the measured equivalent depth was at the surface of the mouse holder. The detected IR dose in EP protons was 5.896 Gy (RBE). The dose-averaged LET (LET_d_) was used to assess the EP (LET_d_ = 0.8 keV/µm) and SOBP (LET_d_ = 2.6 keV/µm) (Figure 1E).

Collectively, these results confirm the successful establishment of early brain injury models following EP and SOBP irradiation.

### 3.2. Comparison of Radiation-Induced Acute Brain Response in SOBP and EP Segment Irradiation

**Figure 2:**
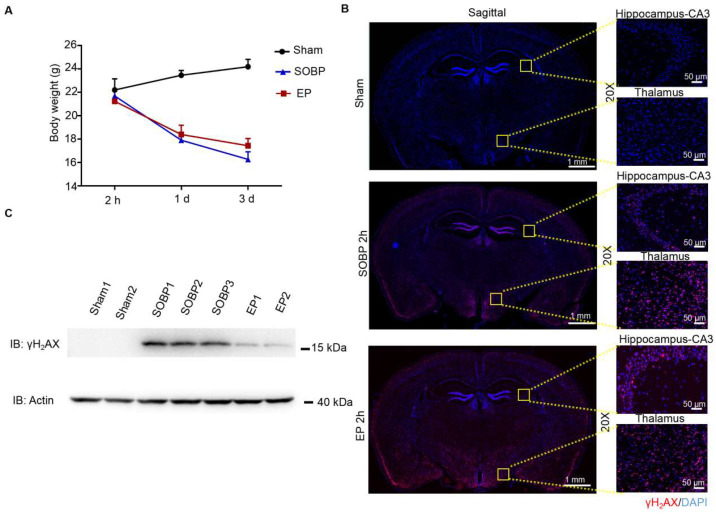
Brain damage detection under SOBP and EP proton irradiation. (**A**) Weight of mice at several time points after IR (Black = Sham group, Blue = SOBP group, Red = EP group). (**B**) Representative fluorescence images of γH2A.X staining in different regions of mice. The scale bar for the sagittal slice of the brain and enlarged pictures is 1 mm and 50 μm, respectively. Nuclei were stained with DAPI (blue signal). (**C**) Immunoblot of γ-H2AX in different groups.

To assess general physiological responses to irradiation, body weight was recorded before irradiation and at 2 h, 1 day, and 3 days post-irradiation. The SOBP group exhibited significantly greater weight loss than the EP group (Figure 2A). DNA damage was evaluated via immunofluorescence and protein expression of γ-H_2_AX. At 2 h post-irradiation, SOBP irradiation induced more intense γ-H_2_AX red fluorescence in the nuclei of the hippocampus and thalamus (Figure 2B), as well as elevated γ-H_2_AX protein levels, compared with EP irradiation (Figure 2C).

**Figure 3:**
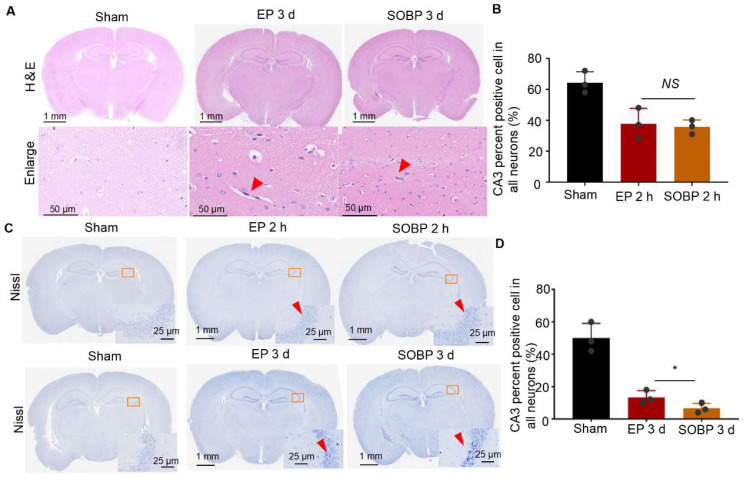
Physiopathological detection in brain tissues under SOBP and EP proton irradiation. (**A**) Representative images of H&E staining in the sham, EP, and SOBP groups 1 day after proton irradiation. Arrowheads indicate eosinophils. Scale bar = 1 mm/50 μm. (**B**) Nissl-stained images showing percentage of positive cells in the CA3 region 2 h after SOBP or EP proton irradiation. (**C**) Representative Nissl-stained images 2 h and 3 days after SOBP or EP proton irradiation. Orange boxes indicate the CA3 region. Scale bar = 1 mm/25 μm. (**D**) Nissl-stained images showing percentage of positive cells in the CA3 region 3 days after SOBP or EP proton irradiation. *NS*: Student’s *t*-test with *p* > 0.05, *: Student’s *t*-test with *p* < 0.05.

Histological assessment was performed using H&E and Nissl staining. H&E staining revealed characteristic eccentric nuclei and pronounced eosinophilic cytoplasm in reactive astrocytes in both groups (Figure 3A). Nissl staining was employed to assess neuronal distribution in the hippocampus 2 h and 3 days post-irradiation. While initial neuronal damage was observed in both groups at 2 h, more severe neuronal loss was evident in the SOBP group than in the EP group at 3 days post-irradiation (Figure 3B–D and Figure S2).

Collectively, these findings demonstrate that SOBP proton irradiation induces more severe early radiobiological effects in the brain than EP irradiation.

### 3.3. Metabolomic Profiling of the Hippocampus Under SOBP or EP Segment Irradiation

**Figure 4:**
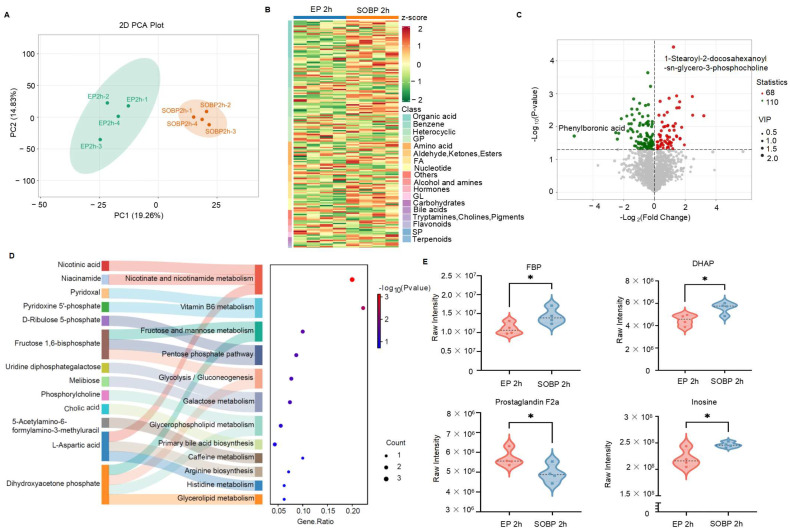
Analysis of metabolic profiling between SOBP and EP in brain samples 2 h after proton irradiation. (**A**) Metabolic score plot of PCA in brain samples. (**B**) Heatmap showing the abundance of significant metabolites identified in both groups, with the associated class. (**C**) Volcano plots of differential metabolites between SOBP and EP in brain samples. Red and green indicate upregulated and downregulated metabolites in the SOBP group, respectively. (**D**) Sankey diagram depicting the interrelationships between metabolic KEGG pathways and gene ratios. (**E**) Expression of differential metabolites (FBP, DHAP, PGF2α, and inosine) in SOBP and EP brain samples. *: Student’s *t*-test with *p* < 0.05.

Principal component analysis (PCA) demonstrated that the metabolites within each group clustered together and that there was a distinct separation between groups. In addition, 19.26% and 14.83% of the variance were explained by principal components 1 and 2, respectively (Figure 4A). Heatmap analysis further demonstrated significant alterations 2 h post-irradiation between the SOBP and EP (Figure 4B).

To identify differentially expressed metabolites (DEMs), we compared metabolite abundances between EP and SOBP groups using a threshold of VIP > 1 and *p* < 0.05 (Figure 4C). A total of 178 DEMs (68 upregulated, 110 downregulated) were identified 2 h post-irradiation (Figure 4C). Notably, among these DEMs, a higher proportion of “organic acids and their derivatives” was upregulated than downregulated in response to SOBP irradiation.

Sankey diagram analysis revealed that nicotinic acid and niacinamide were key regulators in nicotinate and nicotinamide metabolism, while pyridoxal and pyridoxine 5’-phosphate were involved in vitamin B6 metabolism. Notably, fructose-1,6-bisphosphate (FBP), L-aspartic acid, and dihydroxyacetone phosphate (DHAP) were found to regulate more than three signaling pathways (Figure 4D). Among the DEMs, FBP, DHAP, prostaglandin F2α, and inosine exhibited particularly high fold changes (Figure 4E).

At 3 days post-irradiation, PCA showed slight differences in the EP and SOBP groups based on metabolite features (Figure S3A). Heatmap analysis revealed a minor shift in metabolic responses, and only 87 DEMs (45 upregulated, 42 downregulated) were identified during this period (Figure S3B,C).

These data indicate that SOBP proton irradiation induces more extensive metabolic perturbations at 2 h than at 3 days post-irradiation compared with EP.

### 3.4. Proteomic Profiling of the Hippocampus Under SOBP or EP Segment Irradiation

**Figure 5:**
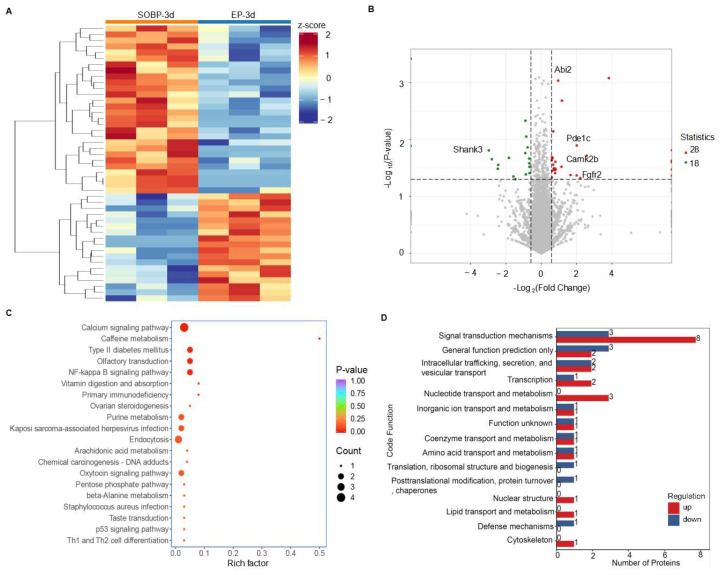
Analysis of proteomic profiles between SOBP and EP in brain samples 3 days after proton irradiation. (**A**) Heatmap showing the abundance of significant proteins identified in both groups. (**B**) Volcano plots of differential proteins between SOBP and EP in brain samples. Red and green indicate upregulated and downregulated metabolites in the SOBP group, respectively. (**C**) Bubble plot visualization of KEGG pathways from DEPs. (**D**) Bar chart using KEGG pathways from DEPs. Red columns refer to upregulated protein numbers in the SOBP group. Blue columns refer to downregulated numbers in the SOBP group.

Proteomic analysis was performed on hippocampal tissues collected at 3 days post-irradiation. Heatmap analysis revealed significant alterations in protein expression between EP and SOBP groups (Figure 5A). Differentially expressed proteins (DEPs) were identified using thresholds of FC ≥ 1.5 or FC ≤ 0.6667 with *p* < 0.05. A total of 46 DEPs (28 upregulated, 18 downregulated) were identified at 3 days post-irradiation (Figure 5B).

KEGG pathway enrichment analysis of the identified DEPs revealed significant enrichment in pathways related to “Calcium signaling pathway”, “AMPK signaling pathway”, “Purine metabolism”, and “Endocytosis” (Figure 5C). Function analysis using eukaryotic orthologous groups (KOG) revealed that DEPs were predominantly involved in “Signal transduction mechanisms”, “Intracellular trafficking, secretion, and vesicular transport”, and “Nucleotide transport and metabolism” (Figure 5D and Table 1).

Thus, these findings suggest that DEPs may contribute to early brain damage through signal transduction-related pathways in the SOBP and EP after 3 days of irradiation.

### 3.5. Integrated Metabolomic and Proteomic Analysis Indicated That Arachidonic Acid Metabolism, Pentose Phosphate Pathway, Purine Metabolism, and Phospholipase D Signaling Pathway Varied Between SOBP and EP Segment Irradiation

**Figure 6:**
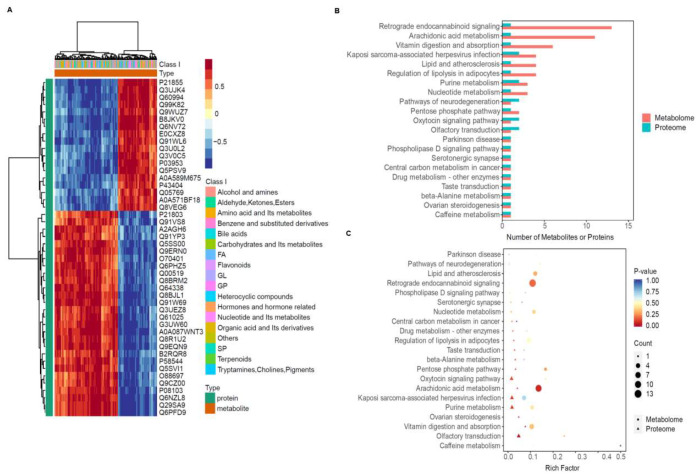
Multi-omics integration of proteomics and metabolomics, and schematic of represented pathways under SOBP and EP proton irradiation. (**A**) Map of multi-omics signatures in clustered images. Columns show selected features; rows show samples. Yellow: mild linkage; red/blue: positive/negative associations. (**B**) Bar chart using co-enriched KEGG pathways from proteomic and metabolomic data. (**C**) Bubble plot visualization of co-enriched KEGG pathways from proteomic and metabolomic data.

Correlation heatmaps were generated based on 178 annotated DEMs (2 h post-irradiation) and 46 DEPs (3 days post-irradiation) (Figure 6A). Integrated analysis identified 21 shared KEGG pathways between the metabolomic and proteomic datasets (Figure 6B). Significantly enriched signaling pathways were revealed via KEGG analysis of the combined metabolome and proteome, including “Retrograde endocannabinoid signaling”, “Arachidonic acid metabolism”, “Pentose phosphate pathway” and “Phospholipase D signaling pathway” (Figure 6B). Among these, “Pentose phosphate pathway,” “Arachidonic acid metabolism,” and “Vitamin digestion and absorption” were found to be significantly enriched in the common KEGG analysis of the metabolomes and proteomes, indicating their critical role in the early brain damage phenotype of murine models caused by SOBP proton irradiation (Figure 6C).

**Figure 7:**
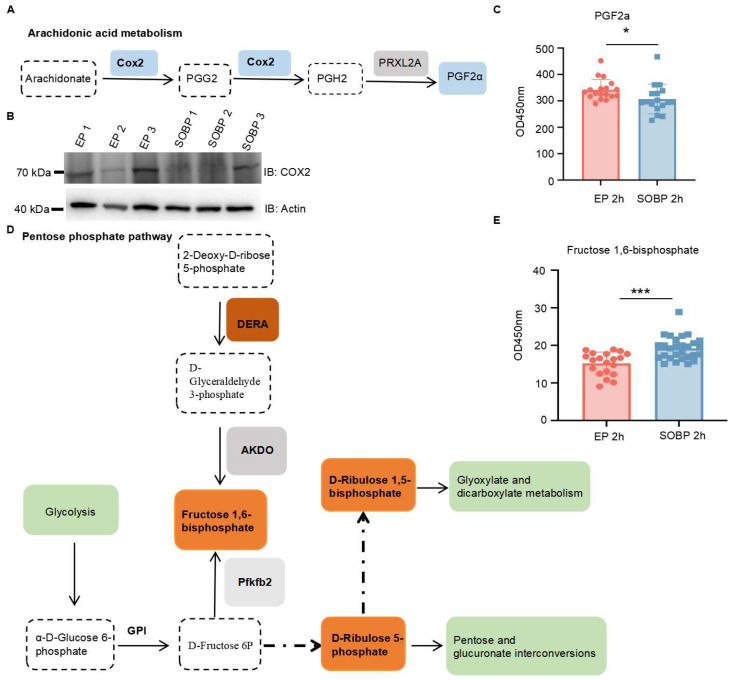
Schematic of related pathways (arachidonic acid metabolism and pentose phosphate pathway) based on multi-omics and validation of specific protein expression under SOBP or EP proton irradiation. (**A**) Schematic of arachidonic acid metabolism based on conjoint analysis of multi-omics in proton irradiation. The blue boxes show decreased proteins/metabolites in the SOBP group compared to the EP group. The gray boxes show detected but insignificantly varied proteins between the SOBP and EP groups. (**B**) Immunoblot of COX2 in the SOBP and EP groups. (**C**) Serum content of PGF2α in the SOBP and EP groups. (**D**) Schematic of pentose phosphate pathway based on conjoint analysis of multi-omics in proton irradiation. The green boxes show biological processes, and the orange boxes show increased metabolites in the SOBP group compared to the EP group. The gray boxes show detected but insignificantly varied proteins between the SOBP and EP groups. Solid arrows indicate the next step; hollow arrows indicate omitted intermediate steps. (**E**) Serum content of FBP in the SOBP and EP groups. *: Student’s *t*-test with *p* < 0.05. ***: Student’s *t*-test and *p* < 0.001.

**Figure 8:**
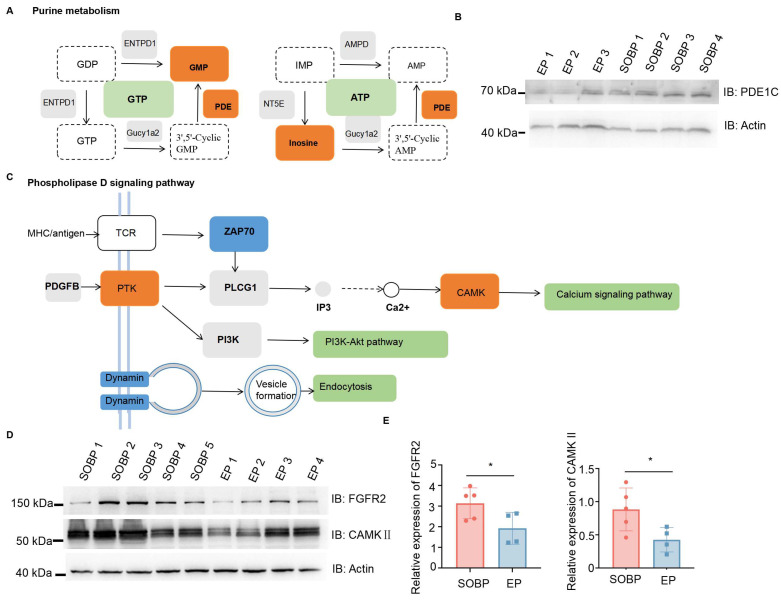
Schematic of related pathways (purine metabolism and phospholipase D signaling pathway) based on multi-omics and validation of specific protein expression under SOBP or EP proton irradiation. (**A**) Schematic of purine metabolism based on conjoint analysis of multi-omics in proton irradiation. The green boxes show biological processes. The blue boxes show decreased proteins/metabolites and the orange boxes show increased proteins/metabolites in the SOBP group compared to the EP group. The gray boxes show detected but insignificantly varied proteins between the SOBP and EP groups. (**B**) Immunoblot of PDE1C in the SOBP group compared to the EP group. (**C**) Schematic of the phospholipase D signaling pathway based on conjoint analysis of multi-omics in proton irradiation. The green boxes show biological processes. The blue boxes show decreased proteins/metabolites and the orange boxes show increased proteins/metabolites in the SOBP group compared to the EP group. The gray boxes show detected but insignificantly varied proteins between the SOBP and EP groups. Solid arrows indicate the next step; hollow arrows indicate omitted intermediate steps. (**D**) Immunoblot of FGFR2 and CAMK II in the SOBP group compared to the EP group. (**E**) Quantification of protein level is shown in (**D**). *: Student’s *t*-test with *p* < 0.05.

**Figure 9:**
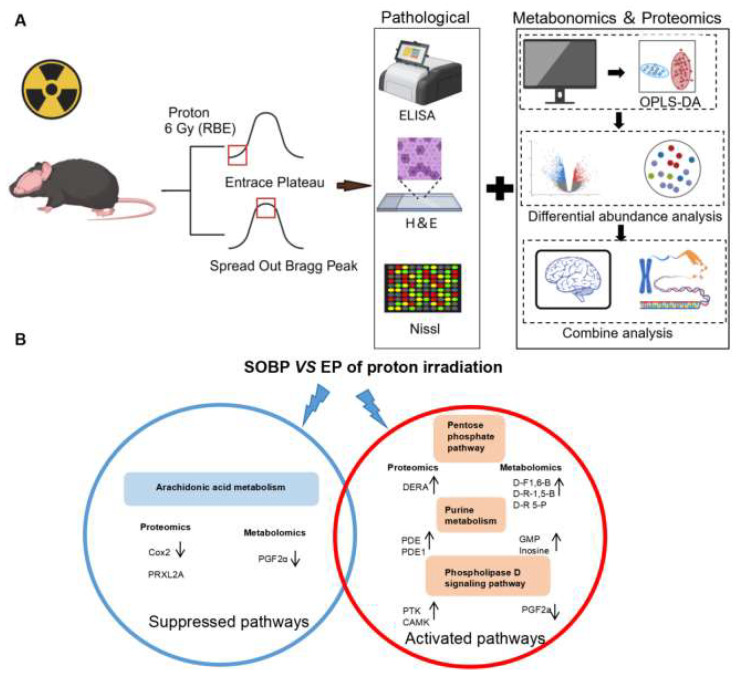
Schematic diagram of mouse model with early brain injury and multi-omic changes and related pathways under IR. (**A**) Schematic diagram of the mouse modeling and analysis workflow. (**B**) Multimodal analysis of differential metabolic processes between the EP and SOBP groups.The blue boxes show decreased biological processes and the orange boxes show increased biological processes in the SOBP group compared to the EP group.

To validate the findings from the integrated analysis, we performed targeted validation of key proteins and metabolites within the identified pathways. For arachidonic acid metabolism, we measured the serum prostaglandin F2α (PGF2α) concentration at 2 h post-irradiation and Cox2 protein expression at 3 days post-irradiation in male and female mouse. Serum PGF2α concentration is downregulated two hours after SOBP compared to EP proton irradiation, as shown in Figure 7A,C and Figure S5A. In comparison to EP proton irradiation, the Cox2 protein is likewise downregulated three days after SOBP (Figure 7B and Figures S4A and S5B,C). For the pentose phosphate pathway, we measured the serum fructose-1,6-bisphosphate (FBP) concentration. FBP was significantly elevated in the SOBP group compared with the EP group (Figure 7D–E and Figure S5D), consistent with the metabolomic findings. For purine metabolism, we validated PDE1C protein expression. PDE1C was significantly upregulated in the SOBP group compared with the EP group (Figure 8A,B and Figures S4B and S5B), confirming the proteomic results. For the phospholipase D signaling pathway, we validated two downstream proteins: calcium/calmodulin-dependent protein kinase II (CAMK II) and fibroblast growth factor receptor 2 (FGFR2). Both proteins were significantly upregulated in the SOBP group compared with the EP group (Figure 8D,E and Figure S5B). Figure 9 illustrates the research framework and the differential metabolic pathways between the EP and SOBP groups.

Collectively, these validated proteins and metabolites, which are involved in signal transduction and metabolic processes, may contribute to the more severe brain damage observed after SOBP proton irradiation.

## 4. Discussion

In this study, we systematically compared the radiobiological effects of the entrance plateau ($EP,{LET}_{d}=0.8\;keV/µm$) and the center of the spread-out Bragg peak ($SOBP,\;{LET}_{d}=2.6\;keV/µm)$ in a murine model of whole-brain irradiation at an equivalent physical dose of 6 Gy (RBE). Our integrated metabolomic and proteomic analyses revealed distinct molecular signatures between EP and SOBP irradiation, with SOBP inducing more extensive metabolic perturbations at 2 h post-irradiation and more pronounced proteomic alterations at 3 days post-irradiation, correlating with greater neuronal loss in the hippocampus. Notably, SOBP irradiation differentially modulated key pathways—upregulating the pentose phosphate pathway, purine metabolism, and phospholipase D signaling, while suppressing arachidonic acid metabolism—a pattern not previously described for proton beam segment-specific effects in the brain. These findings demonstrate that LET heterogeneity along the proton beam path, even at equivalent physical doses, translates into biologically distinct outcomes in normal brain tissue, underscoring the inadequacy of physical dose metrics alone for predicting proton-induced normal tissue toxicity. Collectively, our multi-omics characterization of segment-specific radiobiological responses provides a molecular framework for understanding the differential brain injury profiles associated with EP and SOBP irradiation and identifies potential targets along the beam path that may inform strategies to mitigate radiation-induced brain injury in proton therapy.

Our time-course analysis showed that metabolic perturbations were most pronounced at 2 h post-irradiation, with the EP and SOBP profiles diverging significantly at this early time point. Among the differentially expressed metabolites, PGF2α was significantly downregulated in the SOBP group, which is consistent with evidence linking COX2 reduction to neurological disorders [23,24], suggesting that suppressed arachidonic acid metabolism contributes to SOBP-associated brain injury. Conversely, FBP plays crucial physiological roles in the respiratory, reproductive, and digestive systems [25]. FBP was markedly elevated in SOBP, together with increased DERA and other identified enzymes (GPI, PFKFB2, and AKDO), indicating activation of the pentose phosphate pathway and crosstalk with Glycolysis, Glyoxylate and dicarboxylate metabolism [26]. The purine metabolites GMP and inosine were also upregulated, implicating purine metabolism dysregulation in the early radiation response. In line with our findings, purine nucleoside metabolites are significant biomolecules that are involved in a number of physiological activities, including signal transduction, energy metabolism, and neurotransmission [27,28]. Given that many proteins remained unchanged at 2 h, we focused the proteomic analysis on 3 days post-irradiation. FGFR2, a receptor tyrosine kinase (RTK), can activate many pathways, including the RAS-RAF-MAPK pathway, the PI3K-AKT pathway, the PLCγ-PKC pathway, and the JAK-STAT pathway [28]. CaMK2, the essential mediator of learning and memory, contributes to peripheral and central neuropathic pain [29]. These proteins were significantly elevated in SOBP, implicating phospholipase D and calcium signaling pathways. These findings suggest that restoring PGF2α or inhibiting DERA, PDE, and CAMK2 may represent novel strategies to mitigate radiation-induced brain injury.

Meanwhile, the biological damage at the EP is less extensive than at the SOBP. Proton irradiation may be due to the proton’s physical characteristics. Our finding of differential dose–response between the entrance (0.8 keV/μm) and SOBP (2.6 keV/μm) is consistent with in vitro and in vivo evidence. Particularly, the degree and complexity of biological damage rise with increasing ionization density and LET from the EP to SOBP proton paths, as evidenced by a higher incidence of γH2AX lesions. Experimental data by Guan et al. showed that as LET_d_ increased from 0.9 keV/μm to 3 keV/μm, there was a 4–15% increase in RBE with regard to clonogenic cell survival [30]. Saager et al. demonstrated a clear leftward shift in the dose–response curve for the grade 2 paresis in the mouse spinal cord at the 2.7 keV/μm position compared with the 1.4 keV/μm position, reflecting greater biological effectiveness at the higher LET [31]. Histological examination confirmed necrosis and cellular contraction, accompanied by pronounced neuron loss under the SOBP segment. The current protective strategy in proton therapy mainly focuses on inhibitors, such as EZH2 selective degrader [32], which cannot completely inhibit the progression of normal tissue toxicity. Any clinical biomarker to identify such injuries would be a major development, as early diagnosis of neurological alterations is challenging. Our findings revealed that early intervention may prevent late-stage damage.

As a result, our study shows that the variations in damage caused by proton beam segments with varying LET occur at both at the molecular and physical levels. These include upregulation of the pentose phosphate pathway, purine metabolism, and phospholipase D signaling, and suppression of arachidonic acid metabolism. The temporal divergence between metabolomic (2 h) and proteomic (3 days) changes suggests an early intervention window. The novelty of this study is underscored by three key contributions: (i) a systematic in vivo comparison of EP and SOBP irradiation at equivalent physical dose, isolating LET-driven biological effects; (ii) an integrated multi-omics framework that captures the temporal hierarchy of metabolic and proteomic responses along the proton beam path; and (iii) the identification of a coordinated pathway-level signature—encompassing arachidonic acid metabolism, pentose phosphate pathway, purine metabolism, and phospholipase D signaling—that distinguishes SOBP from EP effects and reveals actionable targets for early intervention. These findings provide a molecular rationale for developing segment-specific protective strategies to improve the therapeutic ratio of proton therapy.

However, this study has several limitations: (1) in-depth exploration of the mechanisms underlying those signaling pathways should be further verified, and (2) the short observation period limits the representation of late irradiation damage.

## 5. Conclusions

Our findings underscore the need for early detection of proton-induced brain toxicity and demonstrate that the higher-LET SOBP segment causes more severe damage than the EP segment. Targeting these dysregulated multi-omics pathways (restoring PGF2α or inhibiting DERA, PDE, and CAMK2) may offer a promising strategy for mitigating radiation-induced brain injury during proton therapy.

## Figures and Tables

**Table 1:** Specific DE genes/DE proteins of signal transduction according to GO conjoint analysis.

DEP	EP 3d	SOBP 3d	KEGG Pathways	*p* Value
Abi2	0.23 ± 0.03	0.45 ± 0.03	Regulation of the actin cytoskeleton	0.0009
Cd72	0.07	0	B cell receptor signaling pathway	0.000378997
Camk2b	0.11 ± 0.05	0.25 ± 0.02	ErbB signaling pathway; Calcium signaling pathway; cAMP signaling pathway; Neurotrophin signaling pathway;	0.030053523
Pde1c	0.17 ± 0.01	0.25 ± 0.03	Purine metabolism; Calcium signaling pathway;	0.023174977
Farp2	0.1 ± 0.03	0.18 ± 0.03	Rap1 signaling pathway;	0.038899902
Fgfr2	0.04 ± 0.07	0.2 ± 0.05	EGFR tyrosine kinase inhibitor resistance; MAPK signaling pathway; Ras signaling pathway; Rap1 signaling pathway; Calcium signaling pathway; Endocytosis; PI3K-Akt signaling pathway;	0.047609622
Stk16	0.15 ± 0.04	0.25 ± 0.04		0.032065175
Shank3	0.09 ± 0.03	0.01 ± 0.02	Glutamatergic synapse	0.022148261
Scube1	0	0.06 ± 0.02		0.023509937
Hck	0	0.32 ± 0.12	Chemokine signaling pathway; Fc gamma R-mediated phagocytosis;	0.043274697
Cacna1e	0.34 ± 0.04	0.22 ± 0.05	MAPK signaling pathway; Calcium signaling pathway;	0.030134227

## Data Availability

The original contributions presented in this study are included in the article/Supplementary Materials. Further inquiries can be directed to the corresponding authors.

## Supplementary Materials

The following supporting information can be downloaded at: https://www.mdpi.com/article/10.3390/cells15151382/s1, Figure S1: Positioning and dose verification of proton irradiation; Figure S2: Nissl staining of brain tissue slices under SOBP and EP proton irradiation; Figure S3: Analysis of metabolic profiling between SOBP and EP in brain samples 3 days after proton irradiation; Figure S4: Quantification of COX2 and PDE1C protein level is shown in Figure 7 and Figure 8. Figure S5: The validation of key proteins and metabolites within the identified pathways in female mouse.
