# Supplementary material for: Distinct Metabolomic and Proteomic Signatures of Early Brain Damage Triggered by the Entrance Plateau Versus the Spread-Out Bragg Peak of Proton Radiation
Source: Cells. 2026 Jul 30;15(15):1382. doi: 10.3390/cells15151382 (PMC13463892; doi:10.3390/cells15151382)
Supplement: Supplementary file 1 [file cells-15-01382-s001.zip › cells-4380036-supplementary.pdf]

Supplementary Materials for

Distinct Metabolomic and Proteomic Signatures of early brain damage triggered by the Entrance plateau versus the Spread-out Bragg peak of proton radiation

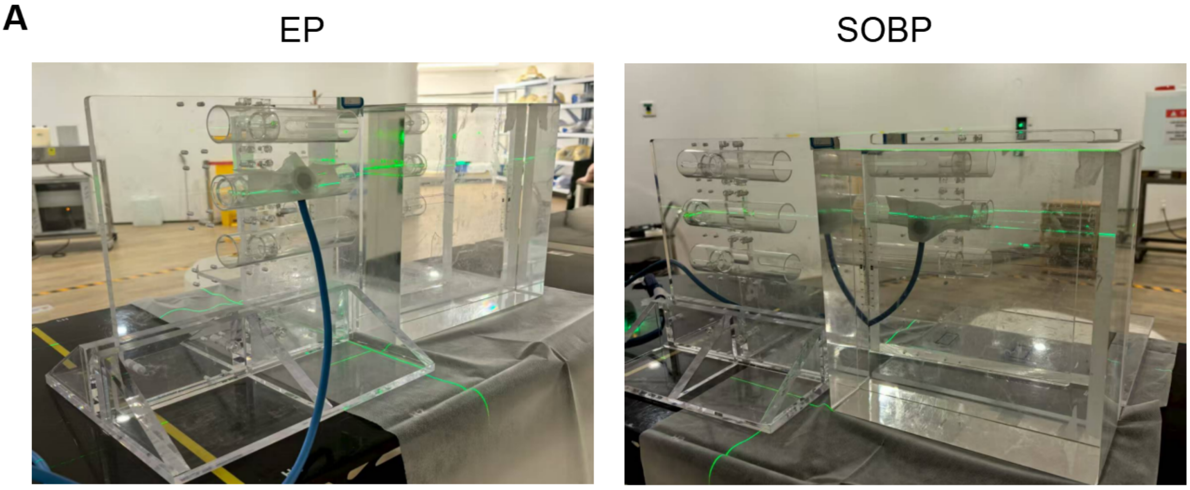

Figure S1. Positioning and dose verification of proton irradiation. (A) The dose detection for the applied proton plan in the mouse model.

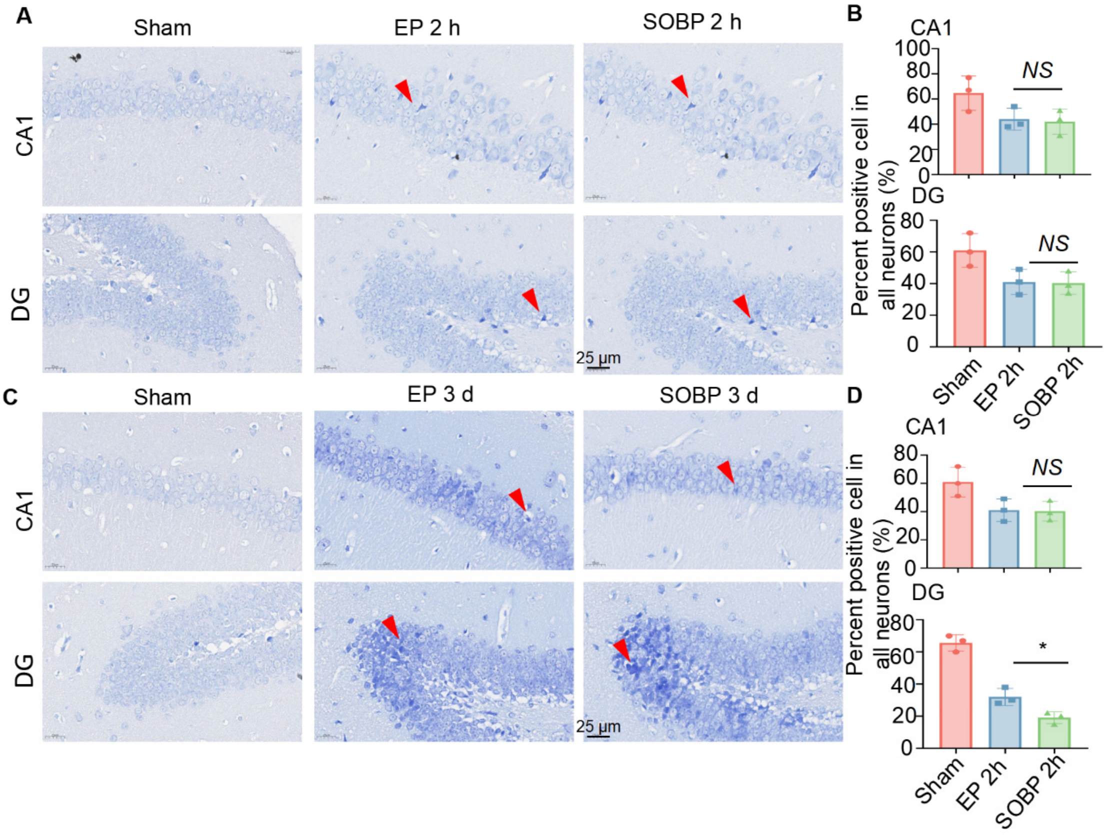

Figure S2. Nissl staining of brain tissue slices under SOBP and EP proton irradiation. (A) Representative images of Nissl staining in sham, EP, and SOBP 2 hours post-proton irradiation. Scale bar = 25 μm. (B) Percentage of positive cells in the CA1 and DG regions 2 hours post SOBP or EP proton-treated mice by Nissl staining. (C) Representative images of Nissl staining 3 days post SOBP or EP proton-treated mice. Scale bar = 25 μm. (D) Percentage of positive cells in the CA1 and DG regions 2

hours post SOBP or EP proton-treated mice by Nissl staining. *: Student's t-test and p < 0.05.

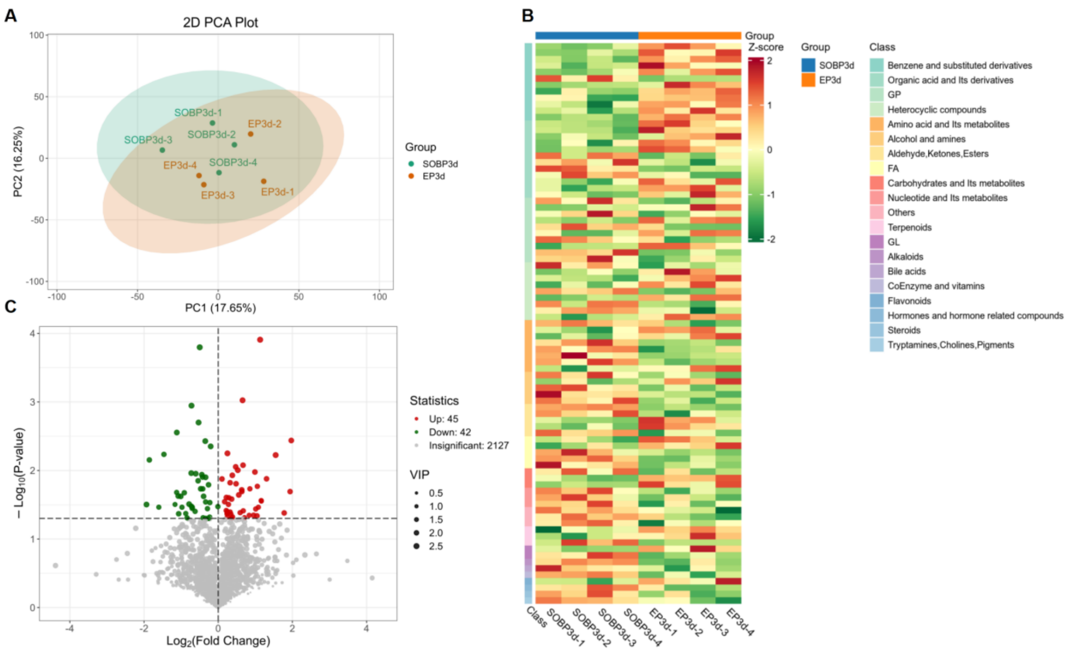

Figure S3. Analysis of metabolic profiling between SOBP and EP in brain samples 3 days after proton irradiation. (A) Metabolic score plot of PCA in brain samples. (B) Heatmap showing the abundance of significant metabolites identified in both groups, with the associated class. (C) Volcano plots of differential metabolites between SOBP and EP in brain samples. The red represents upregulated metabolites in the SOBP group. The green represents downregulated metabolites in the SOBP group.

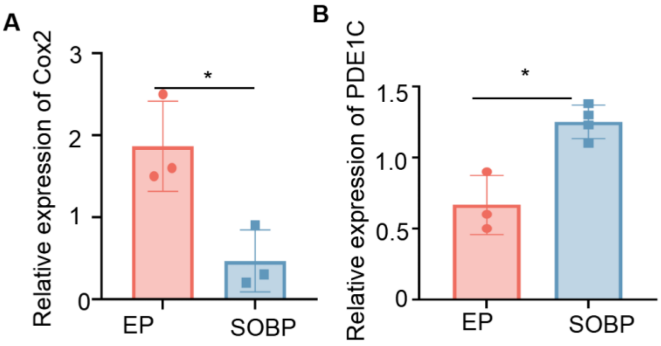

Figure S4. Quantification of COX2 and PDE1C protein level is shown in Fig 7 and 8. (A) Quantification of Cox2 protein level is shown in Fig 7B. (B) Quantification of PDE1C protein level is shown in Fig 8B.*: Student's t-test and $p < 0.05$.

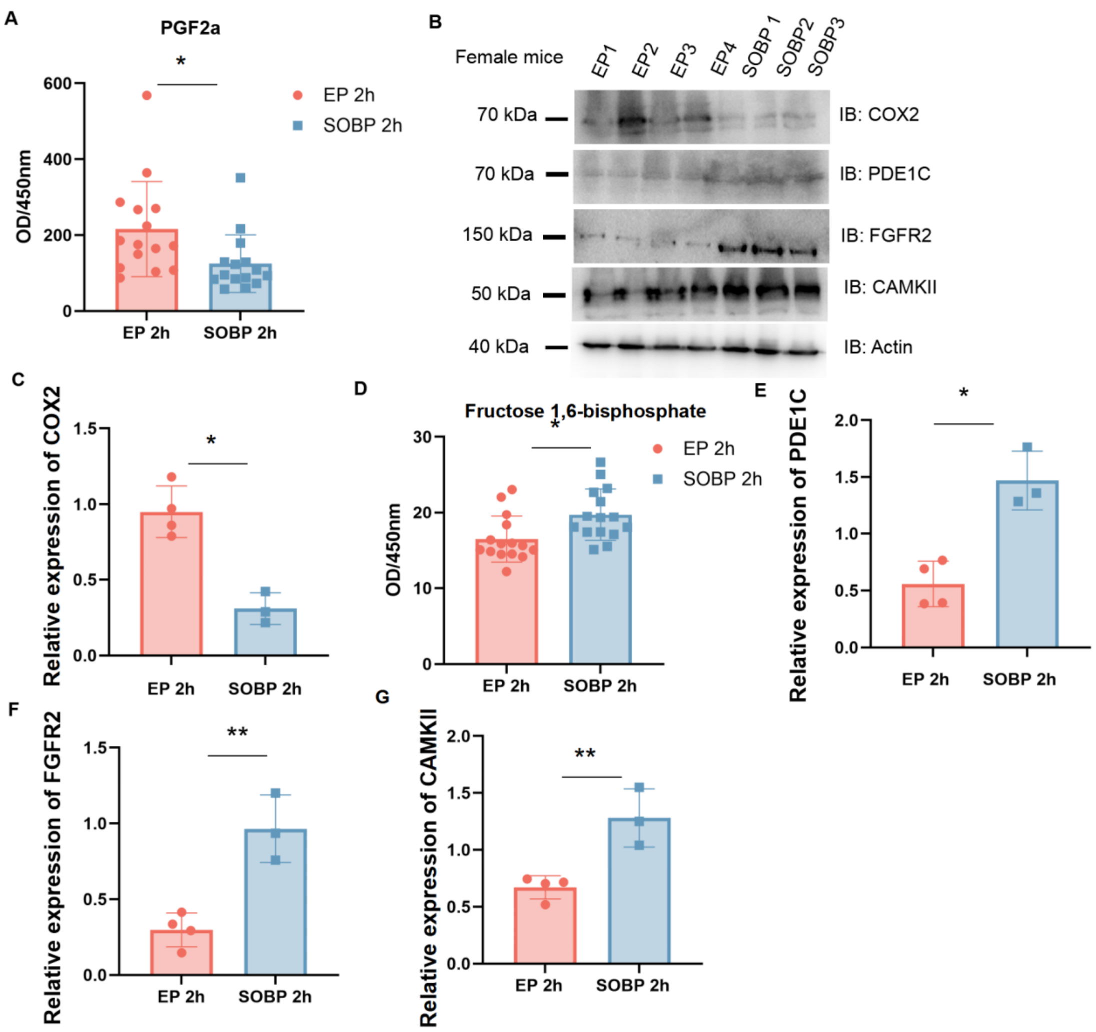

Figure S5. The validation of key proteins and metabolites within the identified pathways in female mouse. (A) Serum content of PGF2α in the SOBP and EP groups. (B) Immunoblot of COX2/PDE1C/FGFR2/CAMKII in the SOBP and EP groups. (C) Quantification of Cox2 protein level. (D)Serum content of FBP in the SOBP and EP groups. (E) Quantification of PDE1C protein level. (F)Quantification of FGFR2 protein level. (G)Quantification of CAMKII protein level. *: Student's t-test and $p < 0.05$. **: Student's t-test and $p < 0.01$.
